# Association between empagliflozin exposure and skeletal muscle degeneration in patients with heart failure

**DOI:** 10.1186/s12872-026-06003-4

**Published:** 2026-08-31

**Authors:** Tatsuya Nishikawa, Nobuto Nakanishi, Kayo Osawa, Junko Mizuno, Yuko Ono, Ayaki Shirahata, Takumi Hirabayashi, Mikio Shiba, Ryohei Fujiwara, Itsuki Kanemitsuya, Yuki Hoshi, Hodaka Noda, Hibiki Kadohara, Yuya Ohga, Motohiro Shingu, Yuto Osumi, Kenta Ishibashi, Toshimitsu Ishii, Mana Hiraishi, Mitsuo Kinugasa, Yasutaka Hirayama, Toshio Shimokawa, Masafumi Matuo, Koichi Tamita

**Affiliations:** 1 Department of Cardiovascular Medicine, Akashi Medical Centre, 743-33 Yagi, Ohkubo-cho, Akashi, Hyogo 674-0063 Japan; 2 Division of Disaster and Emergency Medicine, Department of Surgery Related, Kobe University Graduate School of Medicine, Kobe, Japan; 3 Faculty of Health Sciences, Kobe Tokiwa University, Kobe, Japan; 4 Department of Rehabilitation, Konan Medical Centre, Kobe, Japan; 5 Division of Rehabilitation Medicine, Kobe University Hospital, Kobe, Japan; 6 Cardiovascular Division, Hyogo Prefectural Nishinomiya Hospital, Nishinomiya, Japan; 7 Department of Rehabilitation, Akashi Medical Centre, Akashi, Japan; 8 Department of Biostatistics, School of Medicine, Wakayama Medical University, Wakayama, Japan; 9 Graduate School of Science, Technology and Innovation, Kobe University, Kobe, Japan

**Keywords:** Titin, Heart failure, SGLT2 inhibitor, Quality of life, Frailty

## Abstract

**Background:**

Sodium–glucose co-transporter 2 inhibitors (SGLT2i) have become essential in the management of heart failure. Their prognostic benefits are consistent across older adults, frail, and underweight patients, but there may be potential adverse effects on the skeletal muscle.

**Methods:**

In this prospective single-centre observational study, 117 patients with stable congestive heart failure received 10 mg of empagliflozin daily between February 2023 and October 2024. At baseline and again after 6 months, we measured the following nutritional, frailty, and quality-of-life indices (GNRI, CONUT, mini nutritional assessment (MNA), KCCQ-12, Barthel index, clinical frailty scale) and urinary titin N-fragment (U-titin), a biomarker of skeletal muscle degradation.

**Results:**

After exclusion, 93 patients (median age 79 years; 40% women) were analysed. Baseline U-titin showed weak correlations with GNRI, MNA, and KCCQ-12 scores (*p* < 0.05). Median U-titin increased significantly from 1.7 (IQR 1.1–3.5) at baseline to 2.4 (IQR 1.3–4.2) pmol/mg Cr at 6 months (p = 0.040). The GNRI, MNA, KCCQ-12, body mass index, and NT-proBNP levels improved significantly, whereas the CONUT, Barthel index, and frailty scores remained unchanged. Left ventricular ejection fraction ≥50% independently predicted  ≥50% U-titin increase (OR 3.31, 95% CI 1.23–9.52, *p* = 0.017).

**Conclusions:**

Baseline U-titin levels seem to reflect nutritional and functional status in patients with heart failure. U-titin levels increased during empagliflozin treatment, suggesting a possible association with skeletal muscle degradation.

**Supplementary Information:**

The online version contains supplementary material available at https://doi.org/10.1186/s12872-026-06003-4.

## Introduction

Sodium–glucose co-transporter-2 inhibitors (SGLT2i) have become valuable drugs for the treatment of patients with heart failure. Notably, it improves prognoses for both heart failure with preserved ejection fraction (HFpEF) and heart failure with reduced ejection fraction (HFrEF) [1, 2]. Although it was developed for diabetes, it has been widely accepted as a drug for heart failure and chronic kidney disease. The mechanism of lowering the level of blood sugar is the inhibition of glucose reabsorption in the proximal renal tubule. Theoretically, SGLT2i lowers plasma glucose levels by increasing urinary glucose excretion, thereby reducing the overall glycemic load. Therefore, in older adults or frail patients who might have insufficient calorie intake, the use of SGLT2i might have a negative nutritional effect.

The benefits on clinical outcomes, including cardiovascular death, hospitalisation for heart failure, and quality of life (QOL), have been shown to be consistent across different patient subgroups, including those defined by frailty, age, and body mass index [3–5]. However, real-world data indicate that older patients are less likely to receive SGLT2 inhibitors, possibly because of treatment inertia and concerns regarding comorbidities and tolerability, despite guideline recommendations irrespective of age [6]. This discrepancy highlights the gap between clinical evidence and real-world practice.

Skeletal muscle dysfunction is increasingly recognised as a central component of the pathophysiology of heart failure, contributing to exercise intolerance, frailty, and reduced QOL, and has been reported to play a role across both the early and chronic stages of the disease [7, 8]. Given the potential concerns regarding nutritional status and muscle health in older patients, we aimed to evaluate changes in skeletal muscle–related parameters during SGLT2 inhibitor treatment.

Here, we investigated the evident markers for nutrition, frailty, QOL, and muscle degradation by urinary titin N-fragment. Titin is the largest protein located in the muscle sarcomere. Urinary titin N-fragment has become measurable and has gained attention as a novel non-invasive biomarker for muscle weakness and atrophy in patients with acute and chronic illnesses [9–14]. We prospectively investigated the changes in skeletal muscle–related biomarkers during empagliflozin treatment by measuring urinary titin N-fragment.

## Methods

### Study design and population

This was a single-centre prospective observational study. This study was approved by the Akashi Medical Centre Ethics Review Board (Approval Number: 2022-27), and all procedures were conducted in accordance with the Declaration of Helsinki. Written informed consent was obtained from all participants. We recruited 117 consecutive patients with heart failure who provided signed informed consent. The sample size was determined pragmatically based on feasibility, targeting approximately 100 patients after exclusion; therefore, this study should be considered exploratory. All patients had received empagliflozin for the first time between February 2023 and October 2024 at our hospital and were clinically stable outpatients or in a stable state immediately before discharge from hospital treatment for heart failure. The recruited patients were followed up for 6 months. Exclusion criteria were those who withdrew consent, those lost to follow-up, those who stopped taking empagliflozin after any adverse events, and those who had worsened activities of daily living following cardiac surgery. Adverse events included urinary tract and genital infections, dizziness, lightheadedness, and metabolic complications such as ketoacidosis and hypoglycaemia, as well as any condition that interfered with the patient’s daily life.

## Measurement

The primary endpoint was the change in U-titin levels at 6 months. The secondary endpoints included changes in nutritional status, QoL, frailty indices, and cardiopulmonary exercise testing (CPET) parameters. Urinary titin N-fragment (U-titin) was obtained at initiation and 6 months after the initiation of SGLT2i. Urinary titin N-fragment was measured using a #29,501 N-Titin assay kit (Immuno-Biological Laboratories Co., Ltd., Gunma, Japan) and adjusted by urinary creatinine concentration, which was measured using DICT-500 (Creatinin Assaykit, QuantiChrom, BioAssay Systems, CA, USA) following the manufacturer’s protocol. Absorbance was measured at 450 nm using an iMark™ Microplate Absorbance Reader (Bio-Rad Laboratories, Inc., Hercules, California, USA). An enzyme-linked immunosorbent assay was performed by trained investigators who were blinded to the study design.

The patients’ nutritional state was assessed by questionnaires such as mini nutritional assessment (MNA) [15], geriatric nutritional risk index (GNRI) [16], and controlling nutritional status (CONUT) score [17], which were calculated for each patient at the initiation of empagliflozin and 6 months later. For QOL and frailty assessment, we used the Kansas City Cardiomyopathy Questionnaire-12 (KCCQ-12) [18], Barthel index [19], , and clinical frailty scale [20] with the same timing. All instruments were developed previously and were not modeified in the present study. The KCCQ-12 is an especially well-regarded score for symptoms and QOL in patients with heart failure. Within the KCCQ-12 scoring system, the KCCQ12-CS (clinical summary score) and KCCQ12-OS (overall summary score) were used for assessment. The KCCQ-12 questionnaire was used under the licence obtained from Outcomes Instruments LLC. The cut-off values were set in the present study as GNRI <92 (major nutrition-related risk); [21] MNA <17 (protein-calorie malnutrition); [15] CONUT >4 (moderate to severe undernutrition); [22, 23] Barthel index <85 (incomplete independence); and [24] clinical frailty scale >5 (moderate to severe frailty) [25].

CPET was performed for those who were eligible and tolerant to exercise using an ergometer (*n* = 74). The peak VO_2_ (mL/kg/min), VE/VCO_2_ slope, and ΔVE/ΔWR data were obtained from the CPET. CPET parameters, including the peak VO_2_ and VE/VCO_2_ slope, were assessed according to established guidelines and standard methodologies [26]. ΔVE/ΔWR was defined as the relationship between minute ventilation (VE) and work rate (WR) during exercise testing. Additionally, we assessed past medical history and medication at the initiation of empagliflozin. The sample size was set to meet an arbitrary number of approximately 100 patients after exclusion.

All patients underwent standard clinical evaluation, including electrocardiography and echocardiography, within a clinically relevant time frame. Left ventricular ejection fraction (LVEF) was assessed, and heart failure severity was classified according to the New York Heart Association (NYHA) functional classification.

## Outcomes and definitions

As outcomes of the 6-month treatment with empagliflozin, we assessed urinary titin N-fragment (U-titin), body mass index (BMI) (kg/m^2^), KCCQ12-CS, KCCQ12-OS, MNA, GNRI, CONUT, Barthel index, and clinical frailty score, CPET data, and NT-proBNP (pg/mL). A universal cut-off value of U-titin has not yet been established, but the normal range of U-titin in healthy adult volunteers has been reported as 2.1 (95%CI, 1.2–2.6) pmol/mg Cr [11, 27]. All past reports used U-titin as corrected by urinary creatinine, as shown in the unit. We therefore considered a notable increase in U-titin to be an ‘increase of ≥50% from baseline,’ which could make the normal range abnormal. The group of patients who satisfied the definition was named ‘with U-titin increase,’ and others were named ‘without U-titin increase.’ As a sensitivity analysis, we also analysed with the endpoint of U-titin, defined as ‘increase ≥30% from baseline.’

### Statistical analysis

Nonparametric continuous data are presented as median [interquartile range, IQR], and differences were compared using the Wilcoxon rank sum test. Categorical variables are presented as n (%), and differences were compared using two-tailed Fisher’s exact test. For the comparison of markers or scores from baseline to 6 months, continuous data were analysed using the Wilcoxon signed-rank test, while categorical data were analysed using the McNemar test. Multivariate logistic regression analysis was performed using backward stepwise regression analysis with Akaike information criterion (AIC). Analysis of the trend of continuous variables was performed using the Jonckheere–Terpstra test. For all analyses, a *p* value of < 0.05 was considered to indicate statistical significance. Statistical analyses were performed using GraphPad Prism 9 software (GraphPad Software, CA, USA), EZR version 1.68 (https://www.jichi.ac.jp/saitama-sct/SaitamaHP.files/statmedEN.html), and JMP version 11.0 (SAS Inc., Tokyo, Japan).

## Results

### Patient characteristics

**Fig. 1:**
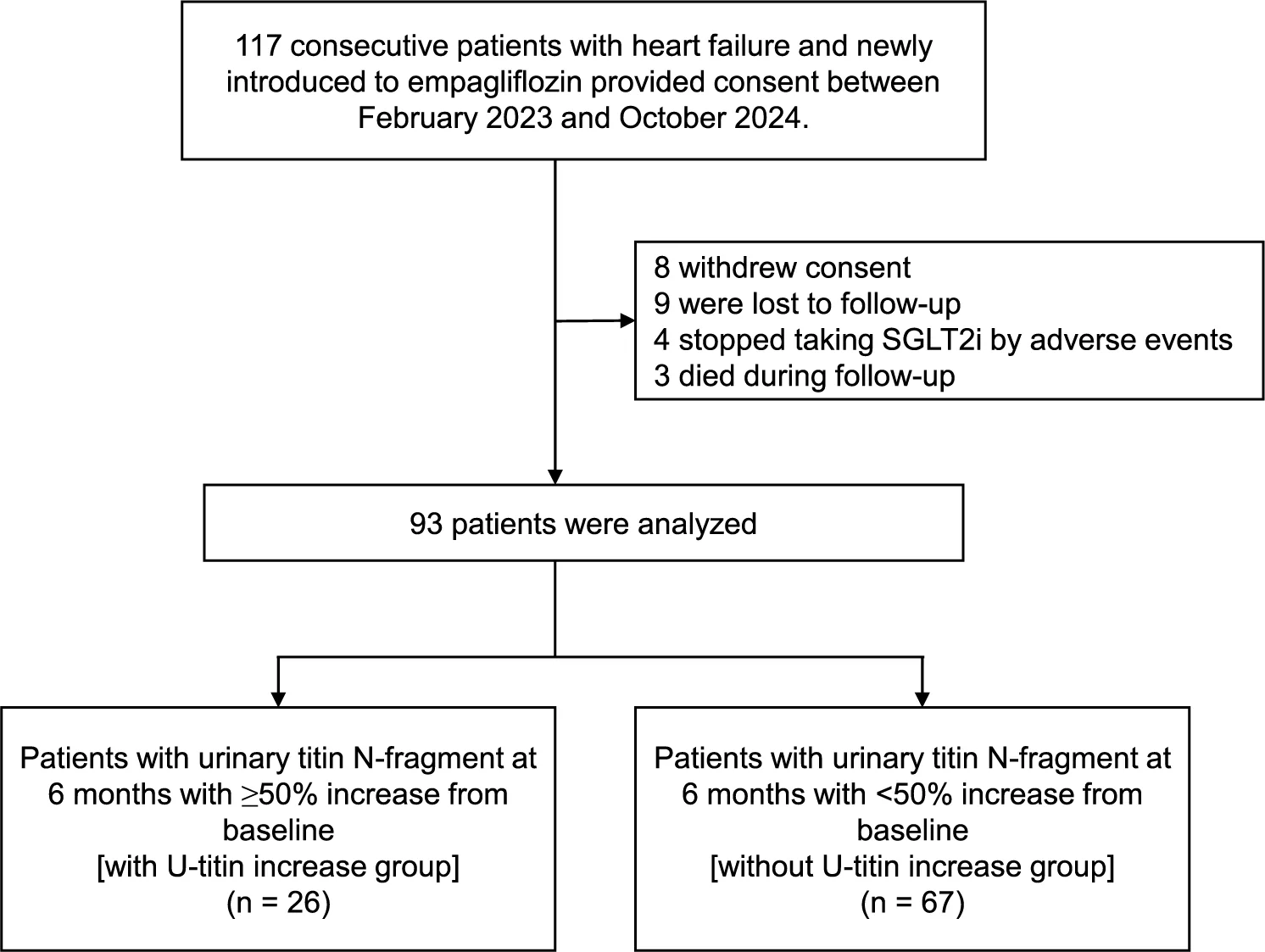
Study flow chart. The study flowchart is shown in figure. SGLT2i, Sodium–glucose co-transporter 2 inhibitors

In the present study, 93 patients were analysed after excluding 24 patients (Fig. 1). Among the excluded patients, 4 patients were excluded due to adverse events, including 2 cases of urinary tract infection and a case of fatigue and genital pruritus. The median age was 79 years (IQR, 72–83), and 37 (40%) were women (Table 1). The median baseline BMI was 22.8 kg/m^2^ (IQR, 19.8–26.2). As echocardiographic data, the median baseline LVEF was 48.7% (IQR, 36.0–62.0). The prevalence of LVEF ≥ 50% was 46%. The median baseline HbA1c level was 6.2% (IQR, 5.8–6.2%). The prevalence of chronic kidney disease was 77%. The medications at baseline were as follows: beta-blocker, 84%; angiotensin-converting enzyme inhibitor (ACEi)/angiotensin Ⅱ receptor blocker (ARB), 33%; angiotensin receptor neprilysin inhibitor (ARNI), 55%; and Mineralocorticoid Receptor Antagonist (MRA), 66%.

**Table 1:** Baseline characteristics of the total cohort and of patients with or without an increase in urinary titin N-fragment levels at 6 months from baseline

	Total cohort (*N* = 93)		Without U-titin increase (*n* = 67)		With U-titin increase (*n* = 26)		*p* value
Age	79	[72-83]	79	[72-84]	79	[70-82]	0.774
Female	37	(40)	23	(34)	14	(54)	0.102
BMI, kg/m^2^	22.8	[19.8–26.2]	22.8	[20.8–26.2]	22.4	[18.6–26.5]	0.411
DM	20	(22)	12	(18)	8	(31)	0.260
HT	59	(63)	45	(67)	14	(54)	0.242
Dyslipidemia	40	(43)	27	(40)	13	(50)	0.486
COPD	6	(6)	5	(7)	1	(4)	1.000
CKD	72	(77)	51	(76)	21	(81)	0.785
Peripheral artery disease	10	(11)	8	(12)	2	(8)	0.720
Hospitalised for HF within the previous 12 months	3	(3)	1	(1)	2	(8)	0.188
History of more than 2 hospitalisation for HF	2	(2)	1	(1)	1	(4)	0.483
Echocardiography							
LVEDD, mm	48.1	[41.9–54.1]	49.1	[43.7–54.2]	44.8	[40.5–53.2]	0.061
LVESD, mm	35.6	[29.4–43.2]	36	[31.0–45.0]	33.7	[25.6–42.0]	0.078
LVEF, %	47.8	[35.4–60.8]	44.9	[35.3–58.6]	54.2	[40.3–65.8]	0.109
LVEF ≥ 50%	43	(46)	27	(40)	16	(62)	0.104
LA diameter, mm	38.8	[34.5–44.7]	39.4	[34.2–45.1]	36.7	[35.0–39.7]	0.287
AS > grade 2	1	(1)	1	(1)	0	(0)	1.000
AR > grade 2	4	(4)	1	(1)	0	(0)	0.573
MR > grade 2	12	(13)	9	(13)	3	(12)	1.000
Laboratory data							
Total protein, g/dL	6.8	[6.4–7.2]	6.8	[6.3–7.2]	6.8	[6.6–7.2]	0.440
Albumin, g/dL	3.8	[3.5–4.1]	3.8	[3.4–4.0]	3.9	[3.5–4.1]	0.295
Creatinine, mg/dL	1.1	[0.9–1.3]	1.1	[1.0–1.3]	1.0	[0.9–1.2]	0.231
HbA1c, %	6.2	[5.8–6.5]	6.2	[5.8–6.5]	6.1	[5.7–6.3]	0.284
NT-proBNP, pg/mL	861	[399–2118]	870	[428–2188]	676	[378–2256]	0.710
Haemoglobin, g/dL	13.6	[12.5–14.3]	13.7	[13.0–14.4]	12.7	[11.9–13.9]	0.017
Haematocrit, %	41.4	[38.5–44.8]	42.4	[40.0–45.6]	39.6	[36.4–42.1]	0.013
CRP	0.17	[0.08–0.61]	0.29	[0.09–1.20]	0.13	[0.06–0.29]	0.047
Medication							
beta-blocker	78	(84)	56	(84)	22	(85)	1.000
ACEI/ARB	31	(33)	18	(27)	13	(50)	0.049
ARNI	51	(55)	41	(61)	10	(38)	0.064
Statin	43	(46)	31	(46)	12	(46)	1.000
MRA	61	(66)	46	(69)	15	(58)	0.340
Loop diuretics	48	(52)	36	(54)	12	(46)	0.645
Vasopressin V2 receptor inhibitor	9	(10)	7	(10)	3	(12)	1.000
Thiazide diuretics	3	(3)	3	(4)	0	(0)	0.557
Outpatient cardiac rehabilitation	21	(23)	7	(27)	14	(21)	0.585
NYHA classification							0.393
II	74	(80)	55	(82)	19	(73)	
III	19	(20)	12	(18)	7	(27)	

*BMI* Body mass index, *DM* Diabetes mellitus, *HT* Hypertension, *COPD* Chronic obstructive pulmonary disease, *CKD* Chronic kidney disease, *LVEDD* Left ventricular end-diastolic diameter, *LVESD* Left ventricular end-systolic diameter, *LA* Left atrium, *AS* Aortic stenosis, *AR* Aortic regurgitation, *MR* Mitral regurgitation, *NT-proBNP* N-terminal pro–B-type natriuretic peptide, *CRP* c-reactive protein, *ACEI* Angiotensin-converting enzyme inhibitor, *ARB* Angiotensin receptor blocker, *ARNI* Angiotensin Receptor-Neprilysin Inhibitor, *MRA* Mineral corticoid receptor antagonist

## Baseline U-titin and nutritional, QOL, and frailty assessment

**Fig. 2:**
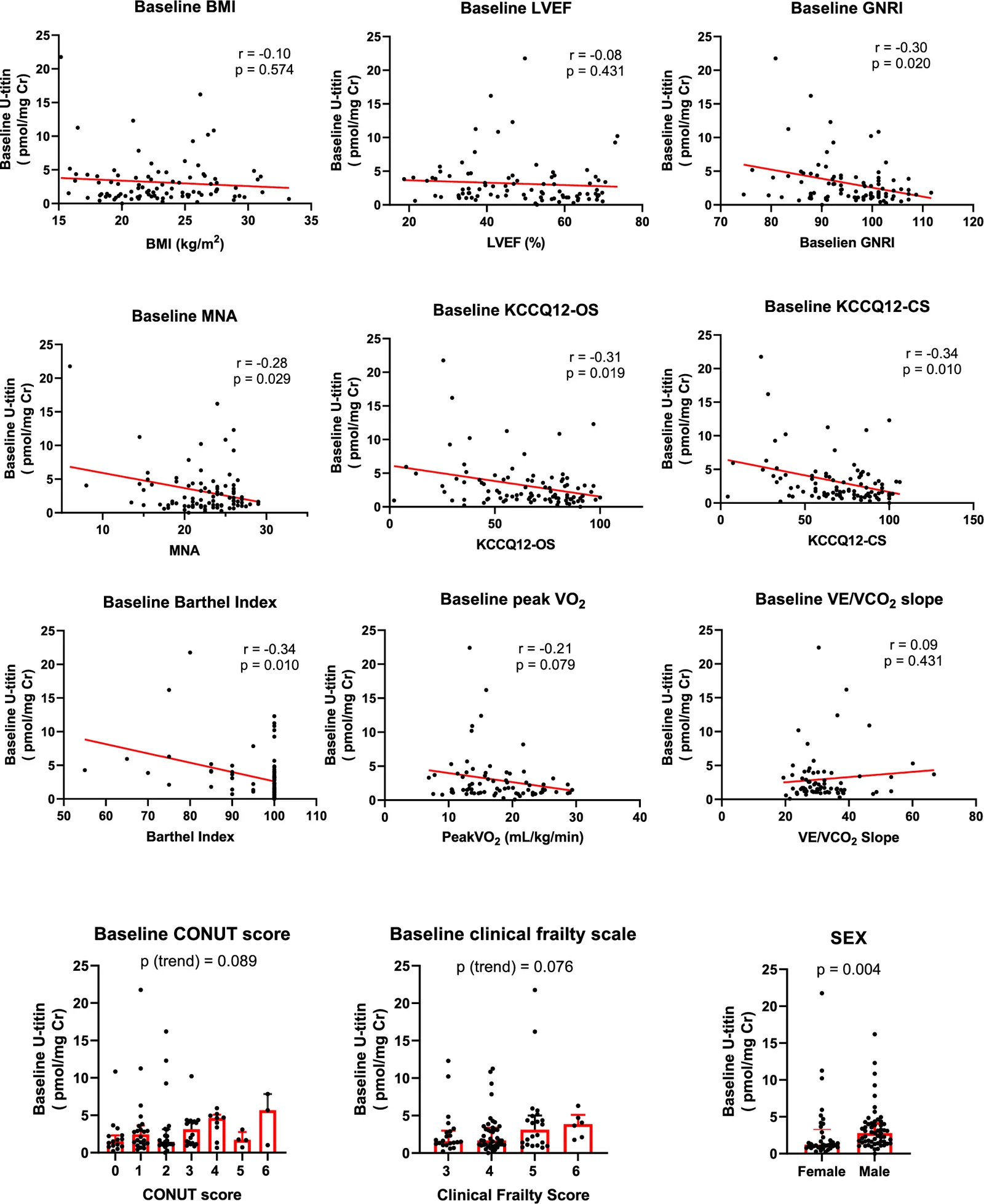
The relationship between baseline U-titin and clinical markers. *P* (trend) was calculated using the Jonckheere–Terpstra test. The bar graph shows the median and IQR

We assessed the relationship between baseline U-titin and baseline BMI, LVEF, GNRI, MNA, KCCQ12-OS, KCCQ12-CS, Barthel index, CONUT score, clinical frailty scale, and sex (Fig. 2). Slight but significant correlations to baseline U-titin were shown in GNRI (*r* = -0.30, *p* = 0.020), MNA (*r* = -0.28, *p* = 0.029), KCCQ12-OS (*r* = -0.31, *p* = 0.019), KCCQ12-CS (*r* = -0.34, *p* = 0.010), and Barthel index (*r* = -0.34, *p* < 0.001). However, BMI, LVEF, Peak VO_2_ (*n* = 74), VE/VCO_2_ slope (*n* = 73), CONUT score, and clinical frailty scale had no significant correlation with baseline U-titin. Women had significantly higher baseline U-titin levels than men (*p* = 0.004).

## Changes in outcomes by 6 months of empagliflozin treatment

The main outcome of the study was the level of U-titin N-fragment as a marker of skeletal muscle degradation [9, 24]. The baseline characteristics and differences between those with and without U-titin increase are shown in Table 1. Among the total cohort, 26 of 93 patients were categorised into the group with increased U-titin levels. There were slight but significant differences in LVEF, albumin level, and haemoglobin level (*p* < 0.05). The differences in the markers of nutrition, QOL, and frailty between the two groups are shown in Table 2. No significant differences were observed for any of the variables.

**Table 2:** Differences in baseline nutritional status, quality of life, frailty scores, and cardiopulmonary exercise testing (CPET) data between patients with and without a defined increase in urinary titin N-fragment levels at 6 months from baseline

	Without U-titin increase (*n* = 67)		With U-titin increase (*n* = 26)		*p* value
Nutritional score					
GNRI	97	[91–101]	98	[87–103]	0.653
GNRI < 92	21	(31)	9	(35)	0.807
CONUT score	2	[1–3]	2	[1–3]	0.785
CONUT score > 4	7	(10)	1	(4)	0.435
MNA total	23.5	[20.5–25.5]	22.8	[19.8–26.0]	0.966
MNA total < 17	7	(10)	3	(12)	1.000
Activity or symptomatic score					
KCCQ12-OS	68	[47–84]	70	[51–85]	0.761
KCCQ12-OS < 50	17	(25)	6	(23)	1.000
KCCQ12-CS	72	[54–86]	73	[60–88]	0.681
KCCQ12-CS < 50	15	(22)	4	(15)	0.573
Barthel Index	100	[100–100]	100	[95–100]	0.623
Barthel Index < 85	4	(6)	3	(12)	0.395
Clinical Frailty scale ≥ 5	20	(30)	8	(31)	1.000
CPET data					
Peak VO_2_, mL/kg/min (*n* = 51 vs. 23)	17.0	[13.6–22.2]	17.2	[13.3–20.4]	0.551
Peak VO_2_ <14 mL/kg/min (*n* = 51 vs. 23)	17	(33)	8	(35)	1.000
VE vs. VCO_2_ slope (*n* = 50 vs. 23)	29.2	[26.2–33.5]	32.3	[27.8–37.5]	0.167
ΔVO_2_/ΔWR (*n* = 47 vs. 20)	9.8	[8.0–11.3]	9.5	[5.0–11.2]	0.607
BMI, kg/m^2^	22.8	[20.8–26.2]	22.4	[18.6–26.5]	0.411
BMI ≥25 kg/m^2^	43	(64)	18	(69)	0.809

Continuous data was assessed by Wilcoxon rank sum test. Categorical data was assessed by Fisher’s exact test

*GNRI* Geriatric nutritional risk index, *CONUT* Controlling nutritional status, *MNA* Mini Nutritional Assessment, *KCCQ12* Kansas City Cardiomyopathy Questionnaire-12, *CPET* Cardiopulmonary exercise testing

The markers of nutrition, QOL, and frailty at baseline and six months are shown in Table 3. The median level of U-titin was 1.7 pmol/mg Cr (IQR, 1.1–3.5) at baseline and 2.4 pmol/mg Cr (IQR, 1.3–4.2) at 6 months. There was a significant difference in U-titin levels at baseline and at 6 months (Wilcoxon signed-rank test, *p* = 0.040). Notably, there were significant improvements in GNRI (*p* < 0.001), MNA (*p* < 0.001), KCCQ12-OS (*p* < 0.01), KCCQ12-CS (*p* < 0.01), BMI (*p* = 0.005), and NT-proBNP (*p* < 0.001). In contrast, there was no significant change in the CONUT score, Barthel index, or clinical frailty score. Among those who were eligible and tolerant of CPET (*n* = 74), peak VO_2_ also significantly improved (*p* = 0.005). The remaining patients (*n* = 19) were ineligible for CPET due to frailty and/or dementia.

**Table 3:** Differences in outcomes between baseline and 6 months

	At baseline (*N* = 93)			At 6 months (*N* = 93)			*p* value	total *n* analysed
Urinary Titin *N*-fragment, pmol/mg Cr	1.7	[1.1–3.5]	(*n* = 93)	2.4	[1.3–4.2]	(*n* = 93)	0.040	(*n* = 93)
Nutritional score								
GNRI	97	[91–101]	(*n* = 93)	101	[96–104]	(*n* = 92)	< 0.001	(*n* = 92)
GNRI < 92	30	(32)	(*n* = 93)	16	(17)	(*n* = 92)	0.003	(*n* = 92)
CONUT score	2	[1–3]	(*n* = 93)	2	[1–3]	(*n* = 93)	0.467	(*n* = 92)
CONUT score > 4	8	(9)	(*n* = 93)	5	(5)	(*n* = 93)	0.366	(*n* = 93)
MNA total	24	[21–26]	(*n* = 93)	25	[23–27]	(*n* = 93)	< 0.001	(*n* = 93)
MNA total < 17	10	(11)	(*n* = 93)	7	(8)	(*n* = 93)	0.317	(*n* = 93)
Activity or symptomatic score								
KCCQ12-OS	67.7	[48.4–84.1]	(*n* = 93)	84.4	[70.1–93.0]	(*n* = 93)	< 0.001	(*n* = 93)
KCCQ12-OS < 50	19	(20)	(*n* = 93)	13	(14)	(*n* = 93)	0.018	(*n* = 93)
KCCQ12-CS	71.9	[56.3–87.5]	(*n* = 93)	89.6	[74.0–100.0]	(*n* = 93)	< 0.001	(*n* = 93)
KCCQ12-CS < 50	23	(25)	(*n* = 93)	10	(11)	(*n* = 93)	0.020	(*n* = 93)
Barthel Index	100	[98–100]	(*n* = 93)	100	[100–100]	(*n* = 93)	0.862	(*n* = 93)
Barthel Indxe < 85	7	(8)	(*n* = 93)	9	(10)	(*n* = 93)	0.317	(*n* = 93)
Clinical Frailty scale	4	[4–5]	(*n* = 93)	4	[4–5]	(*n* = 93)	0.326	(*n* = 93)
Clinical Frailty scale > 5	28	(30)	(*n* = 93)	26	(28)	(*n* = 93)	0.414	(*n* = 93)
CPET data								
Peak VO_2_, mL/kg/min	17.2	[13.3–22.2]	(*n* = 68)	18.6	[14.7–23.7]	(*n* = 68)	0.009	(*n* = 63)
Peak VO_2_ < 14 mL/kg/min	21	(33)	(*n* = 68)	15	(22)	(*n* = 68)	0.013	(*n* = 63)
VE vs. VCO_2_ slope	30.3	[26.7–36.4]	(*n* = 68)	29.5	[26.0–34.6]	(*n* = 68)	0.270	(*n* = 63)
ΔVO_2_/ΔWR	9.8	[7.6–11.1]	(*n* = 67)	10.0	[7.5–11.5]	(*n* = 67)	0.330	(*n* = 57)
BMI, kg/m^2^	22.8	[19.8–26.2]	(*n* = 93)	22.2	[20.1–25.6]	(*n* = 93)	0.005	(*n* = 92)
BMI ≥ 25 kg/m^2^	61	(66)	(*n* = 93)	63	(68)	(*n* = 93)	0.317	(*n* = 93)
NT-proBNP, pg/mL	861	[399–2118]	(*n* = 93)	488	[266–1104]	(*n* = 93)	< 0.001	(*n* = 93)

Continuous data was assessed by Wilcoxon signed-rank test. Categorical data were assessed by McNemar test

Data of CPET were assessed only for those who have had both baseline and 6-month follow-up test

Since underweight patients may be at a higher risk for skeletal muscle degeneration, we additionally assessed changes in U-titin according to BMI category (<18 kg/m^2^ vs. ≥18 kg/m^2^). Although the BMI <18 kg/m^2^ group consisted of only eight patients, patients with a lower BMI tended to show a greater increase in U-titin after 6 months (Supplementary Fig. 1).

### Contributing factors to the increase in U-titin after 6 months of empagliflozin treatment

The contributing factors of U-titin increase ≥ 50% from baseline to 6 months were assessed using multiple regression analysis. Baseline LVEF ≥ 50%, baseline KCCQ12-CS < 50, and baseline Barthel index < 85 were selected by backward stepwise analysis using AIC for multivariate analysis (Table 4). Only LVEF ≥ 50% was a significant contributing factor (OR, 3.31, 95% CI 1.23–9.52, *p* = 0.017). Even if analysed with variables of peak VO_2_ from CPET data (*n* = 74), the results showed that the factors chosen by backward stepwise regression using AIC were baseline LVEF ≥ 50% (OR, 3.38, 95%CI, 1.26–9.78, *p* = 0.016) and baseline BMI < 25 kg/m^2^ (OR, 1.41, 95% CI, 0.52–4.06, *p* = 0.508), KCCQ12-CS < 50 (OR, 0.35, 95% CI, 0.08–1.26, *p* = 0.113), and baseline Barthel index < 85 (OR, 5.26, 95% CI, 0.81–34.57, *p* = 0.080). LVEF ≥ 50% was consistently the only significant factor (Supplementary Table 1). To validate the analysis, we performed a sensitivity analysis with the endpoint defined as a U-titin increase ≥ 30%, instead of 50%, from baseline to 6 months. Consistently, LVEF ≥ 50% (OR, 5.69, 95%CI, 2.11–16.78, *p* < 0.001) was one of the significant contributing factors (Supplementary Table 2).

**Table 4:** Multivariate analysis for the endpoint of ≥50% increase in U-titin after 6 months

Multivariate analysis				Stepwise analysis by using AIC		
(*N* = 93)	OR	[95% CI]	*p* value	OR	[95% CI]	*p* value
Age ≥ 80	0.77	[0.22–2.51]	0.669			
Female	2.02	[0.66–6.33]	0.216			
LVEF ≥ 50%	3.10	[0.99–10.43]	0.051	3.31	[1.23–9.52]	0.017
BMI ≥ 25 kg/m^2^	1.26	[0.40–4.12]	0.699			
GNRI < 92	1.34	[0.36–4.91]	0.661			
CONUT score > 4	0.48	[0.02–3.72]	0.515			
MNA total < 17	0.59	[0.07–3.73]	0.584			
KCCQ12-OS < 50	2.09	[0.36–4.91]	0.661			
KCCQ12-CS < 50	0.17	[0.01–1.76]	0.14	0.36	[0.08–1.26]	0.114
Barthel Index < 85	5.72	[0.74–46.10]	0.093	5.01	[0.78–32.56]	0.087
Clinical Frailty scale > 5	1.12	[0.29–4.34]	0.864			

*AIC* Akaike Information Criterion

We also assessed the baseline characteristics of patients with LVEF ≥ 50% (Supplementary Table 3). The results show that they are likely to be higher-aged women with lower NT-proBNP levels, which are typical characteristics of HFpEF in Japan. In contrast, there were no obvious differences in the assessment of nutrition, QOL, and frailty. The baseline U-titin level was significantly lower in patients with LVEF ≥50%.

## Discussion

This prospective observational study suggests clinically useful findings in patients with heart failure. First, the baseline urinary titin N-fragment was shown to be significantly related to nutrition, QOL, frailty scores, and sex. Second, we found that the urinary titin N-fragment significantly increased after 6 months of empagliflozin therapy. Furthermore, there was improvement from baseline in most of the nutrition, QOL, and frailty markers, NT-proBNP, and BMI at 6 months. Third, LVEF ≥50% was a significant predictive factor for a ≥50% increase in urinary titin N-fragment from baseline to 6 months.

The baseline level of the U-titin N-fragment was shown to be a potential marker for the baseline overall clinical state of patients with heart failure. The level of urinary titin N-fragment reportedly correlates with physical function, such as the Barthel index and grip strength in older adult patients with mild trauma [28]. Additionally, it is related to sarcopenia [10]. Our study showed significant correlations between baseline U-titin N-fragment and GNRI, MNA, KCCQ-12 score, Barthel index, and sex. This suggests that people with insufficient nutrition and lower QOL with symptomatic heart failure have higher U-titin N-fragment, meaning a potentially higher risk of frailty or sarcopenia [10, 29]. Moreover, the higher U-titin levels observed in women may reflect differences in skeletal muscle mass and urinary creatinine normalisation, since women generally have lower muscle mass and urinary creatinine excretion than men. Higher urinary titin N-fragments are already known to be related to poor outcomes for patients with various diseases. Moreover, nutritional status and KCCQ-12 are known markers for the prognosis of heart failure [30–32]. The relationships between sex and QOL with both HFpEF and HFrEF have previously been reported. In some reports, women had poorer baseline health-related QOL than men [33, 34]. Interestingly, in our study, there was no correlation between age, BMI, and baseline urinary titin N-fragment. Our results are consistent with those of a previous study on dilated cardiomyopathy [9]. Various factors must, therefore, be related to baseline urinary titin N-fragment, meaning that it is a reflection of the overall condition of each patient. Notably, although the correlation coefficients among our markers were modest, the majority of clinical markers demonstrated consistent results. Conclusively, our findings were a new aspect of assessing the prognosis of patients with heart failure; however, they are fairly consistent with the findings of previous reports.

Importantly, the level of urinary titin N-fragment increased after 6 months of empagliflozin treatment, which was inconsistent with our original hypothesis. The patients in our cohort were mostly those who lived independently and were able to visit our hospital as outpatients with or without some support from their families. Actually, 81% of patients in the present study had a baseline Barthel index ≥95. According to our results, even in these patients, skeletal muscle–related parameters worsened during empagliflozin treatment. However, due to the small sample size, the results were not conclusive. Furthermore, this increase in U-titin could have been observed even without the administration of empagliflozin, since our cohort included older adult patients (median age, 79 years). Conversely, nutritional scores and heart failure markers, such as KCCQ-12 and NT-proBNP, improved during empagliflozin treatment. Empagliflozin has solid evidence that both HFrEF and HFpEF can improve the prognosis of patients with heart failure [1, 2]. SGLT2 inhibitors have been shown to significantly improve indicators of QOL, such as KCCQ-OS and KCCS-CS [35, 36]. The observed improvements in these markers may reflect an improvement in heart failure status during treatment rather than a specific effect of empagliflozin.

In the present study, we defined a significant change in U-titin as ≥50% from baseline to 6 months, and 26 out of 93 patients satisfied the criteria. Multivariate regression analysis showed that a baseline LVEF ≥50% was the only significant predictive factor for the defined criteria. LVEF ≥50% is usually considered HFpEF, and the pathophysiology of HFpEF varies between patients. Patients with HFpEF are older and often have other chronic diseases, such as chronic kidney disease, chronic inflammatory disease, or diabetes [37]. Therefore, these patients generally have a higher risk of hospital admission and subsequent potential risk of hospital-acquired disability [38]. Patients with HFpEF reportedly have poorer QOL than those with HFrEF [39]. In a previous report, patients with HFpEF had lower type I fibres, type I to type II fibre ratio, and capillary-to-fibre ratio in the vastus lateralis muscle than healthy controls, which were significantly related to the lower peak VO_2_ [40]. In a study of mice with HFpEF, histological and magnetic resonance imaging analyses showed a reduction in oxidative fibres, type-2 A fibre atrophy, decreased capillary density, an increase in the fibrotic area, and an intermuscular fat/skeletal muscle ratio in the soleus muscle. This might be related to mitochondrial oxidative phosphorylation and the inflammatory response [41–43]. Elsewhere, human patients with HFpEF have lower mitochondrial content and citrate synthase activity [44]. These findings are thought to be consistent with and support our result that LVEF ≥50% is significantly related to an increase in U-titin. However, in the present study, we did not compare patients treated with and without empagliflozin. According to previous reports, our result could be explained as a consequence of the time course of HFpEF, and maybe not the effect of empagliflozin. Skeletal muscle dysfunction is increasingly recognised as a central component of heart failure pathophysiology, contributing to frailty, reduced exercise capacity, and impaired QOL in both acute and chronic heart failure [7, 8]. Therefore, our findings may provide additional insights into the complex relationship between heart failure, skeletal muscle health, and metabolic therapy.

There are some limitations to our study. This was an observational study and featured a comparatively small number of patients. In addition, the number of patients lost to follow-up after inclusion was relatively high (20.5%). There was also no comparison of the effect of empagliflozin with placebo. However, the intake of SGLT2 inhibitors is currently a class I recommendation for both HFpEF and HFrEF, so it is unethical to compare with a placebo. The absence of a control group limits causal inference. Second, no established cut-off value for U-titin currently exists for frailty, sarcopenia, or prognostic events in heart failure. Furthermore, the study did not directly measure muscle mass, strength, or imaging-based muscle composition, which limits the ability to correlate urinary titin changes with functional or structural muscle loss. The relatively short follow-up period may also be insufficient to capture the long-term effects of SGLT2 inhibitor therapy on skeletal muscle. Third, the number of patients who underwent CPET was small. Patients who were able to perform CPET may have had a lower risk of muscle degeneration than those who could not. However, this difference could not be assessed in the present study. Patients with severe frailty or dementia were ineligible for CPET. The inclusion of patients with stable heart failure represents a population at lower risk of myopathy and muscle degeneration compared with those with acute heart failure or recent hospitalisation. However, our results adequately reflect real-world clinical situations. Fourth, there is growing evidence for the use of U-titin as a marker for various diseases and clinical situations. However, evidence in relation to heart failure remains insufficient. In the present study, the threshold of a ≥50% increase in U-titin level was exploratory and not based on validated HF-specific reference values. In addition, relative changes may be influenced by low baseline values, potentially exaggerating small biological changes. Therefore, data interpretation requires caution. Fifth, the exclusion of patients who discontinued empagliflozin due to adverse events may have introduced selection and attrition bias. Therefore, the possibility that skeletal muscle–related deterioration was underestimated cannot be excluded. In addition, our cohort had a very low prevalence of a history of hospitalisation for HF within the previous 12 months and of two or more hospitalisations for HF, which are usually associated with a worse prognosis and greater comorbidity [45]. The majority of our cohort had a stable condition or de novo HF. Sixth, although U-titin was normalised to the urinary creatinine concentration, renal function may still influence urinary titin excretion. This issue may be particularly relevant in patients with HF, older adults, and those with CKD who frequently exhibit impaired renal function. Furthermore, SGLT2 inhibitors may affect renal hemodynamics and eGFR. Therefore, the interpretation of creatinine-normalised U-titin levels should be made cautiously, and the possibility of underestimation of skeletal muscle injury cannot be excluded. Therefore, further large-scale clinical research is needed to confirm the usefulness of U-titin as a prognostic biomarker for heart failure. In contrast, a key strength of our study is the use of a variety of clinical markers (including KCCQ-12, MNA, GNRI, CONUT score, Barthel index, and clinical frailty scale) and the demonstration of consistent findings regarding the effect of empagliflozin in patients with heart failure.

Although definitive conclusions cannot yet be drawn regarding the safety of SGLT2 inhibitors with respect to skeletal muscle, this study provides valuable insights into an area of significant clinical interest. Our findings should be interpreted with caution and require confirmation in larger prospective cohorts.

## Conclusion

In patients with heart failure, elevated urinary titin levels were associated with lower GNRI, MNA, KCCQ-12, and Barthel index scores, as well as higher CONUT and clinical frailty scale scores. Urinary titin levels increased after 6 months of empagliflozin treatment, suggesting a possible association between empagliflozin treatment and skeletal muscle changes across a broad spectrum of patients, including older and frail individuals. However, patients with LVEF ≥ 50% may be at risk of accelerated skeletal muscle degradation during empagliflozin treatment, although a causal relationship could not be established based on the present study design.

## Supplementary Information


Supplementary Material 1.


## Data Availability

The datasets used and/or analyzed during the current study are available from the corresponding author on reasonable request.
