# Supplementary material for: Association between empagliflozin exposure and skeletal muscle degeneration in patients with heart failure
Source: BMC Cardiovasc Disord. 2026 Aug 31;26:759. doi: 10.1186/s12872-026-06003-4 (PMC13527901; doi:10.1186/s12872-026-06003-4)
Supplement: Supplementary file 1 [file 12872_2026_6003_MOESM1_ESM.docx]

**Supplementary Material**

**The Effect of Empagliflozin on Skeletal Muscle Degeneration in Patients with Heart Failure**

Tatsuya Nishikawa^1^ MD, PhD, Nobuto Nakanishi^2^ MD, PhD, Kayo Osawa^3^, PhD, Junko Mizuno^1^ RN, Yuko Ono^2^ MD, PhD, Ayaki Shirahata^4^ PT, Takumi Hirabayashi^5^ PT, PhD, Mikio Shiba^6^ MD, PhD, Ryohei Fujiwara^7^ PT, Itsuki Kanemitsuya^7^ PT, Yuki Hoshi^7^ PT, Hodaka Noda^7^ PT, Yuki Irite^7^ PT, Hibiki Kadohara^1^ MD, Yuya Ohga^1^ MD, Motohiro Shingu^1^ MD, Yuto Osumi^1^ MD, PhD, Kenta Ishibashi^1^ MD, Toshimitsu Ishii^1^ MD, Mana Hiraishi^1^ MD, Mitsuo Kinugasa^1^ MD, PhD, Yasutaka Hirayama^1^ MD, Toshio Shimokawa^8^ MD, PhD, Masafumi Matuo^9^, MD, PhD, Koichi Tamita^1^, MD, PhD,

1) Department of Cardiovascular Medicine, Akashi Medical Center, Akashi, Japan
2) Division of Disaster and Emergency Medicine, Department of Surgery Related, Kobe University Graduate School of Medicine, Kobe, Japan
3) Faculty of Health Sciences, Kobe Tokiwa University, Kobe, Japan
4) Department of Rehabilitation, Konan Medical Center, Kobe, Japan
5) Division of Rehabilitation Medicine, Kobe University Hospital, Kobe, Japan
6) Cardiovascular Division, Hyogo Prefectural Nishinomiya Hospital, Nishinomiya, Japan
7) Department of Rehabilitation, Akashi Medical Center, Akashi, Japan
8) Department of Biostatistics, School of Medicine, Wakayama Medical University, Wakayama, Japan
9) Graduate School of Science, Technology and Innovation, Kobe University, Kobe, Japan

Corresponding Author: Tatsuya Nishikawa, MD, PhD

Department of Cardiovascular medicine, Akashi Medical Center

743-33 Yagi, Ohkubo-cho, Akashi, Hyogo 674-0063, Japan

Tel: 81-78-936-1101; Fax: 81-78-936-7456

E-mail: tatsuya.nishikawa.oc@gmail.com

**Supplementary Fig. 1 The relation between those with low BMI and increase rate of U-titin after 6 months.** **≥**

**The bar shows median and the error bar shows IQR. P value is yielded by Wilcoxon rank sum test.**

**Supplementary Table 1 Multivariate analysis of peak VO₂ for the endpoint of a ≥50% increase in U-titin after 6 months**

| Multivariate analysis |  | | | Stepwise analysis by using AIC | | |
| --- | --- | --- | --- | --- | --- | --- |
| (N = 93) | OR | [95% CI] | *p* value | OR | [95% CI] | *p* value |
| Age ≥80 | 0.83 | [0.19-3.36] | 0.797 |  |  |  |
| Female | 2.15 | [0.58-8.28] | 0.253 |  |  |  |
| LVEF ≥50% | 6.28 | [1.55-30.24] | 0.009 | 3.38 | [1.26-9.78] | 0.016 |
| BMI ≥25 kg/m^2^ | 2.43 | [0.62-10.85] | 0.208 | 1.41 | [0.52-4.06] | 0.508 |
| GNRI <92 | 1.37 | [0.34-5.60] | 0.656 |  |  |  |
| CONUT score >4 | 1.18 | [0.04-16.05] | 0.907 |  |  |  |
| MMA total <17 | 0.66 | [0.05-5.77] | 0.714 |  |  |  |
| KCCQ12-OS <50 | 2.72 | [0.34-21.35] | 0.335 |  |  |  |
| KCCQ12-CS <50 | 0.10 | [0.01-1.18] | 0.068 | 0.35 | [0.08-1.26] | 0.113 |
| Barthel Index <85 | 16.07 | [1.08-27.81] | 0.044 | 5.26 | [0.81-34.57] | 0.080 |
| Clinical Frailty scale >5 | 1.80 | [0.34-10.08] | 0.491 |  |  |  |
| Peak VO_2_ <14 mL/kg/min (n = 74) | 0.67 | [0.17-2.44] | 0.546 |  |  |  |

**Supplementary Table 2 Sensitivity analysis for the endpoint defined as a ≥30% increase in U-titin after 6 months**

| Multivariate analysis |  | | | Stepwise analysis by using AIC | | |
| --- | --- | --- | --- | --- | --- | --- |
| (N = 93) | OR | [95% CI] | p value | OR | [95% CI] | *p* value |
| Age ≥80 | 0.98 | [0.31-3.04] | 0.976 |  |  |  |
| Female | 2.31 | [0.77-7.23] | 0.136 |  |  |  |
| LVEF ≥50% | 5.56 | [1.74-20.22] | 0.003 | 5.69 | [2.11-16.78] | <0.001 |
| BMI ≥25 kg/m^2^ | 0.80 | [0.25-2.45] | 0.69 |  |  |  |
| GNRI <92 | 1.54 | [0.41-6.00] | 0.519 |  |  |  |
| CONUT score >4 | 0.98 | [0.11-6.26] | 0.986 |  |  |  |
| MMA total <17 | 1.6 | [0.23-10.82] | 0.624 |  |  |  |
| KCCQ12-OS <50 | 23.88 | [2.80-543.90] | 0.003 | 16.52 | [2.33-339.86] | 0.004 |
| KCCQ12-CS <50 | 0.01 | [0.00-0.13] | <0.001 | 0.02 | [0.00-0.19] | <0.001 |
| Barthel Index <85 | 3.54 | [0.43-28.15] | 0.23 | 4.06 | [0.63-26.47] | 0.105 |
| Clinical Frailty scale >5 | 1.31 | [0.37-4.75] | 0.678 |  |  |  |

**Supplementary Table 3 Baseline characteristics of patients with LVEF <50% and those with LVEF ≥50%**

| Baseline characteristics | LVEF <50% (n = 50) | | LVEF ≥50% (n = 43) | | *p* value |
| --- | --- | --- | --- | --- | --- |
| Age | 76 | [68-80] | 81 | [76-85] | <0.001 |
| Female | 12 | (24) | 25 | (58) | 0.001 |
| BMI, kg/m^2^ | 22.2 | [19.6-25.8] | 23.2 | [20.3-26.5] | 0.359 |
| DM | 10 | (20) | 10 | (23) | 0.802 |
| HT | 33 | (66) | 26 | (60) | 0.668 |
| Dyslipidemia | 22 | (44) | 18 | (42) | 1.000 |
| COPD | 3 | (6) | 3 | (7) | 1.000 |
| CKD | 36 | (72) | 0.3 | (84) | 0.218 |
| Peripheral artery disease | 7 | (14) | 3 | (7) | 0.331 |
| LVEF, % | 36.0 | [32.2-42.8] | 62.3 | [56.7-68.0] | <0.001 |
| Total protein, g/dL | 6.9 | [6.5-7.2] | 6.7 | [6.3-7.2] | 0.398 |
| Albumin, g/dL | 3.8 | [3.4-4.0] | 3.9 | [3.5-4.1] | 0.119 |
| HbA1c, % | 6.2 | [5.9-6.5] | 6.2 | [5.7-6.5] | 0.743 |
| NT-proBNP, pg/mL | 1217 | [504-2535] | 635 | [328-1616] | 0.052 |
| Hemoglobin, g/dL | 13.8 | [13.0-14.8] | 13.3 | [12.4-13.8] | 0.013 |
| beta-blocker | 45 | (90) | 33 | (77) | 0.097 |
| ACEI/ARB | 14 | (28) | 17 | (40) | 0.275 |
| ARNI | 33 | (66) | 18 | (42) | 0.023 |
| Statin | 23 | (46) | 20 | (47) | 1.000 |
| MRA | 40 | (80) | 21 | (49) | 0.002 |
| Urinary Titin N-fragment, pmol/mg Cr | 2.5 | [1.4-4.1] | 1.5 | [1.0-3.1] | 0.016 |
| GNRI | 94 | [88-101] | 98 | [92-103] | 0.079 |
| CONUT score >4 | 5 | (10) | 3 | (7) | 0.721 |
| MMA total | 24 | [20-25] | 23 | [21-26] | 0.381 |
| KCCQ12-OS | 66.7 | [50.8-81.0] | 74 | [44.8-85.4] | 0.495 |
| KCCQ12-CS | 68.8 | [56.3-86.5] | 76 | [42.7-88.5] | 0.538 |
| Barthel Index | 100 | [95-100] | 100 | [100-100] | 0.196 |
| Clinical Frailty scale >5 | 16 | (32) | 12 | (28) | 0.821 |
| Peak VO_2_, mL/kg/min | 19 | [13.5-23.4] | 16.2 | [13.1-20.3] | 0.142 |
